# Effects of Leisure Obstacles, Job Satisfaction, and Physical and Mental Health on Job Intentions of Medical Workers Exposed to COVID-19 Infection Risk and Workplace Stress

**DOI:** 10.3390/healthcare9111569

**Published:** 2021-11-18

**Authors:** Hsiao-Hsien Lin, Jan-Wei Lin, Chao-Chien Chen, Chin-Hsien Hsu, Bing-Sen Lai, Tzu-Yun Lin

**Affiliations:** 1 School of Physical Education, Jiaying University, Meizhou 514015, China; chrishome12001@yahoo.com.tw (H.-H.L.); benson36566666@gmail.com (B.-S.L.); 2 Center for General Education, National Chi-Nan University, Nantou County 54561, Taiwan; cwlin@ncnu.edu.tw; 3 Department of Leisure and Recreation Management, Asia University, Taichung 41354, Taiwan; peter72@asia.edu.tw; 4 Department of Leisure Industry Management, National Chin-Yi University of Technology, Taichung 404401, Taiwan; 5 Department of Sport Information and Communication, National Taiwan University of Sport, Taichung 404401, Taiwan; lincoach8@gmail.com

**Keywords:** COVID-19, medical workers, leisure obstacles, physical and mental health, willingness to quit

## Abstract

The purpose of this study was to investigate the effects of leisure obstacles, job satisfaction, physical and mental health, and work intentions of medical workers in Taiwan. SPSS 26.0 and AMOS 24.0 statistical software were used to analyze 208 questionnaires by basic statistical tests, *t*-tests, and structural model analysis. Results: Under the epidemic, medical workers were unable to develop job identity due to low promotion opportunities and low job achievement. The lack of recreational exercise skills, time, and information created leisure obstacles. In addition, they were unable to express their true selves freely at work, which led to health problems such as reduced enthusiasm, mental weakness, and emotional irritability. In particular, female medical workers felt more strongly about the issues of leisure obstacles and the intention to stay in their jobs. The study found that the higher their job satisfaction, the higher their intention to stay in the job, while the more pronounced the leisure obstacles and physical and mental health problems, the more pronounced their intention to leave.

## 1. Introduction

Since the outbreak of COVID-19 in China in December 2019, the world has been severely hit by the pandemic [1]. Although the route of infection is clear, the initial asymptomatic patients still cannot be effectively diagnosed [2]. Taiwan had achieved good results in epidemic prevention [3], but the government’s overconfidence led to misjudgment of the situation and poor epidemic prevention decisions, resulting in a major invasion by the mutated virus starting on 13 May 2021 [4]. In addition, Taiwan has not yet implemented universal screening and is caught in a vaccine shortage, with a national vaccination rate of only 3.4% and a Level 3 alert status for the fourth week [5]. The total number of confirmed cases in Taiwan has rapidly accumulated to 12,746, of which 34.8% are young adults and 33.9% are severe cases over 60 years old. Only 1133 of them have recovered and the number of deaths has reached 411 [5,6], which is a serious setback to the effectiveness of epidemic prevention in Taiwan [7].

Despite the setback in epidemic control, adequate medical staff can help the government stabilize the epidemic [8], and professional medical staff can provide high-quality medical services to the public [9]. However, due to the rapid spread of the epidemic, the surge in the number of people in autonomous epidemic prevention, and the outbreak of minor and major infections, the pressure on health care facilities and field workers is escalating rapidly [10,11]. According to statistics, the number of medical workers in 2019 has dropped from 180,153 to 174,468 [12,13], and there has been a wave of departures, although the government has ordered the recruitment of retired medical workers to return to the front line to supplement the workforce [14,15]. Since the epidemic cannot be effectively mitigated, the number of confirmed cases is at least 100 per day [2,5] and there is a shortage of resources for critical care [9,10]. There are currently 11,613 hospitalizations and the number of patients with serious illnesses is increasing rapidly. In addition, there are only a limited number of qualified vaccines with high efficacy [16], and health care workers are not willing to receive the existing vaccines with low protection and high side effects. These problems have led to a significant increase in the workload of medical workers [17] and a resurgence of turnover [18,19]. This is a new crisis for the people of Taiwan, who are in dire need of medical facilities and human resources to combat the spread of COVID-19.

Specialized and delicate workloads, significant medical stress, and risk of infection, and increased physical and mental health burdens are the main reasons for medical workers leaving the profession [9,10,18,19]. Relieving work stress and maintaining physical and mental health is the main focus nowadays. Previous studies have shown that maintaining physical and mental health helps to improve work performance and satisfaction, and increases positive work intentions [20]. Leisure exercise can maintain physical and mental health and improve physical fitness, which can positively help to enhance physical immunity, relieve work stress, and stabilize emotions [21]. It has also been shown that if personal leisure obstacles can be removed, job satisfaction can be increased and work intentions can be further enhanced [22]. However, there are time, space, and equipment constraints for individuals to participate in leisure sports [23], as well as differences in exercise habits [24], and medical work stress due to the epidemic [25]. These are the key factors that prevent people from engaging in recreational sports, resulting in insufficient time to plan recreational sports and, thus, creating leisure obstacles [21]. Therefore, it is proposed in this study that identifying the leisure obstacles and enhancing the willingness of medical workers to engage in leisure sports will facilitate the improvement of physical and mental health, help to regulate workplace stress, and increase the desire to stay in the field.

The high turnover of medical workers is a major crisis in Taiwan [18,19], and improving job satisfaction is an important key in order to stabilize the willingness of staff to stay [24]. Job satisfaction can be explored in terms of personal factors and the work environment [26]. A good work environment can increase job comfort and act as an extrinsic non-reward motivational incentive [27]. Establishing a work environment that meets the needs of the individual is conducive to the development of the individual’s abilities and job requirements [28]. Job satisfaction can be assessed in terms of current conditions such as income, job content, autonomy, peace of mind, respectability, contribution, supportive attitude of supervisor, and work environment [29]. The more the job benefits and conditions meet the needs, the higher the satisfaction [30], while the higher the stress level, the lower the satisfaction [31]. The higher the level of job stress, the lower the job satisfaction or organizational commitment and the more inclined to leave [27]. Therefore, the present study proposes that improving the working environment and feelings of medical workers, enhancing their performance and self-confidence, and increasing their job satisfaction should help to increase their willingness to stay in their jobs.

Good physical and mental health of medical workers can improve concentration, stabilize performance, increase self-confidence, stabilize morale [32], accomplish challenges, and increase willingness to work in the face of difficulties [33]. However, the current pandemic has increased not only heavy workload but also pressure of medical staff, affecting their sleep quality [34], and further seriously endangering their physical and mental health [10,14,15]. Therefore, to maintain the physical and mental health of medical workers, it is important to enhance their ability to face difficulties and increase their willingness to work [29,35]. Therefore, the present study believes that examining the current state of physical and mental health can help to understand the real state of physical and mental health of medical workers under stress, and to understand the impact of physical and mental health maintenance on work intentions, which can help to improve medical workers’ perceptions of the workplace and enhance their work intentions.

Medical workers are one of the most important keys to help the government stabilize the epidemic and restore the health of patients [8,9]. However, medical workers themselves are limited by workload, technical expertise, and medical manpower supply [25], and the current outbreak expansion and infection risk add to the stress of medical workers and endanger their physical and mental health [36]. Moreover, Taiwan is currently facing a crisis of COVID-19 community infection, with a rapid increase in the number of confirmed cases per day and a rise in the number of seriously ill patients, putting medical institutions and staff under tremendous pressure [8,10]. This has dampened the morale of current health care workers, which in turn has shaken their belief in employment and led to resignations [10,11,13,14,15]. It is imperative to find a solution to this problem. Since the COVID-19 epidemic, although studies have been conducted on issues such as leisure obstacles, job satisfaction, physical and mental health, and the desire to stay in the workplace [14,37,38,39]; it is not common to discuss the issues of leisure obstacles, job satisfaction, physical and mental health, and the desire to stay in the workplace at the same time, even with medical workers as the target population. Therefore, the present study was conducted to understand the main reasons that prevented medical workers from engaging in leisure activities, and to explore their perceptions of the current workplace environment and physical and mental health, in order to help understand the workplace perceptions and dilemmas of medical workers in Taiwan. Then, the relationship between the leisure obstacles, job satisfaction, physical and mental health, and work willingness is analyzed to identify the key to improve work intentions. Ultimately, the main goal of the study is to propose solutions to stabilize the work intentions of medical workers and improve the problem of fluctuating medical manpower.

## 2. Literature Review

### 2.1. Leisure Obstacles and the Willingness to Stay in the Workplace

Engaging in recreational sports can maintain physical and mental health, improve physical fitness, and have a positive effect on enhancing physical immunity, relieving work stress, and stabilizing negative emotions [19]. Removing personal barriers to leisure should increase job satisfaction and have a positive impact on increasing willingness to work [20]. However, due to the time, space, and equipment constraints of leisure exercise [21], the differences in individual exercise habits [22], and the medical work pressure caused by the epidemic [23], most people are currently unable to obtain sufficient time for leisure exercise, thus creating leisure obstacles. For Taiwanese medical workers, it is of paramount importance to identify the main factors that hinder the practice of leisure activities in the face of the severe medical environment and work pressure, and to seek solutions to maintain physical and mental health, improve work performance and satisfaction, and increase positive work intentions [18].

The sense of dislike that arises when individuals are prevented from participating in or engaging in leisure activities is known as the leisure obstacle [40]. Moreover, when the threat of an epidemic makes it impossible to engage in leisure activities and recreation on a regular basis [41], it contributes to the negative feelings that occur when people are unable to participate or engage in leisure activities. Research indicates that leisure obstacles arise from intrinsic, interpersonal, structural [42], spatial, and physical [43], as well as time, place, and cost [44] factors. Lack of adequate time, habits, venues, courses, and partners are key barriers to engaging in leisure [45,46]. These include reasons such as lack of family support, poor physical condition, activities requiring more skills, lack of time and money, lack of transportation, lack of physical strength and funds, lack of space, and lack of information. Also, it was found that removing personal leisure obstacles had a positive effect on increasing work intentions [20].

Therefore, the investigators expect to investigate the main reasons that prevent medical workers from engaging in leisure activities, and then to establish the relationship between leisure obstacles and the desire to stay in the workplace; additionally, researchers expect to find solutions to increase the motivation of medical workers to engage in leisure activities, promote their physical and mental health improvement, regulate their workplace stress, and reduce their desire to leave the workplace, which is the first objective of the study.

### 2.2. Job Satisfaction and the Willingness to Stay in the Workplace

The high turnover of medical manpower is a major crisis for Taiwan at present [16,17]. To stabilize employees’ willingness to stay, it is important to improve job satisfaction [22]. Job satisfaction can be explored in terms of personal factors and work environment [24]. A good working environment can enhance the comfort of working and can be an external, non-reward, motivational incentive [25]. Establishing a work environment that meets the individual’s needs is beneficial to the individual’s ability to perform on the job requirements [26]. It was pointed out that job satisfaction can be assessed in terms of current situation such as income, job content, autonomy, stability, respectability, contribution, supportive attitude of supervisor, and work environment [27]. Among them, more details can be obtained by exploring issues such as salary, promotion, freedom of working hours, self-fulfillment, sense of competence, and meaningfulness of work. Research confirms that the more the job benefits and conditions meet the individual’s needs, the higher the satisfaction level [28]. The higher the level of strain, the lower the satisfaction level [29], and the higher the stress, the lower the job satisfaction or organizational commitment, the more employees tend to leave [25].

Therefore, we believe that identifying the negative job perceptions of health workers, improving their performance, self-confidence, and job satisfaction should help improve their job motivation. Therefore, this study was conducted to investigate the perceptions and dilemmas of medical workers about the current workplace situation, and then to confirm the influence of job satisfaction on stabilizing the medical workforce. It is expected that the study will find ways to improve the retention willingness of medical workers and stabilize the workforce, which is the second objective of this study.

### 2.3. Physical and Mental Health and the Willingness to Stay in the Workplace

The increasing severity of the epidemic in Taiwan has added stress to the existing work of medical workers, causing a burden on their physical and mental health and work [10,11,13,14,15]. Scholars believe that assisting health workers with their tasks and solving various work challenges can help them improve their performance, increase self-confidence, and stabilize morale [30]. Maintaining good physical and mental health is the best way to improve concentration and find timely ways to overcome challenges when faced with stress [26,30,31]. More details can be obtained by examining mood, ability, enthusiasm, headaches, mental lassitude, high sensitivity to pain, irregular eating, and irritability. Studies have confirmed that the better the current conditions of physical and mental health of an individual, the better he or she is able to maintain adequate physical and mental conditions to face work and maintain a high willingness to stay at work [27,30,31,32].

Therefore, we believe that examining the current state of physical and mental health of medical workers will help us to understand the negative impact of current stress on physical and mental health, and to understand the impact between the current willingness of medical workers to work, and the degree of change in their physical and mental health. Therefore, the effect of epidemic stress on the current physical and mental health of medical workers was investigated prior to finding out whether there is a correlation between the current physical and mental health and the desire to stay in the workplace, which is the third objective of this study.

## 3. Methods

### 3.1. Research Structure

**Figure 1:**
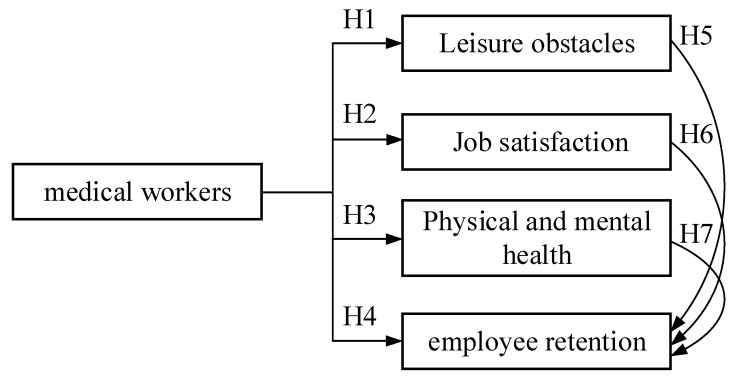
Research Framework.

An adequate number of health care workers can help the government stabilize the outbreak [8], and professional health care workers can provide high-quality medical care to the public [9]. However, the pressure on health care facilities and field workers is rapidly increasing due to the rapid spread of the epidemic, the surge in the number of home quarantine cases, and the outbreak of infected persons with major and minor illnesses [10]. This has led to a significant increase in the workload of medical workers [17] and a resurgence of resignations [18,19]. It also represents a new crisis for the people of Taiwan who are in dire need of medical facilities and human resources to combat the spread of COVID-19. Studies have found that the reasons for medical personnel leaving the workforce can be understood from the perspectives of leisure obstacles [18,20,40,41,42,43,44,45,46], job satisfaction [22,23,24,25,26,27,28,29], physical and mental health, and willingness to stay in the workforce [26,31,32], and can be explored for improvement. Therefore, based on the above descriptions, the objectives of this study, and relevant literature, we proposed a research framework as shown in Figure 1.

### 3.2. Research Hypothesis

The following four research hypotheses were proposed based on the study objectives and related literature:

**Hypothesis 1** **(H1).** *Medical workers have consistent perceptions of leisure obstacles.*

Although the literature has indicated that engaging in leisure activities can improve physical and mental health [19], medical workers are busy with work and shoulder heavy responsibilities [25], their workload has increased due to the impact of the pandemic, [8,10,15,36], and it is believed that this will be one of the main reasons hindering them from engaging in leisure activities. Therefore, it is assumed that medical workers have consistent perceptions of leisure obstacles.

**Hypothesis 2** **(H2).** *Medical workers have consistent perceptions of job satisfaction.*

Saving the dying and the helping the wounded is the calling, duty and mission of medical workers [8,9]. However, in the face of the current attack marked by continuous COVID-19 waves, all the medical workers’ workload and responsibilities have increased greatly, and the pressure on them has also increased [8,10,11,12,13,14,15]. Therefore, it is believed that this would affect their feelings on the work site. So, it is assumed that medical workers have consistent perceptions of job satisfaction.

**Hypothesis 3** **(H3).** *Medical workers have consistent perceptions of current physical and mental health status.*

The medical profession is a kind of high-tech vocation that must be carried out rigorously [9,10,18,19], and it is difficult for medical workers to maintain physical and mental health in a long-term, high-tension working environment [27]. In addition, due to the impact of the pandemic, their workload and pressure have increased, and the relevant labor turnover rate has risen, indirectly resulting in the increase in the workload and pressure of medical workers [31], and affecting their physical and mental health [27]. Therefore, it is believed that currently medical workers’ physical and mental health is put at risk. Therefore, it is assumed that medical workers have consistent perceptions of current personal physical and mental health status.

**Hypothesis 4** **(H4).** *Medical workers have consistent perceptions of the willingness to stay in the workforce.*

Although medical workers in Taiwan generally earn good salaries and have steady jobs [9], as the pandemic has resulted in the increase in their workload and decrease in their willingness to stay, put more pressure on those sticking to their posts, weakening their resolve [27,30,31,32], it is believed that the commitment of medical workers to stick to their posts may not be firm and steadfast anymore. Therefore, it is assumed that the medical workers have consistent perceptions of the willingness to stay in the workforce.

**Hypothesis 5** **(H5).** *Leisure barriers have a significant effect on the willingness to stay in the workforce.*

Leisure is helpful to improve physical and mental health [19], and for medical workers to relieve their huge pressure due to the pandemic and workload [8,10]. However, facilities and space comprise one of the influencing factors that hinder them from engaging in leisure activities. As a result, they cannot get time to engage in leisure activities and effectively relieve the physical and mental pressure from the workplace that affects their willingness to stay in the workforce. Therefore, it is believed that leisure constraints and willingness to stay in the workforce have a significant impact. Therefore, it is assumed that leisure constraints have a significant impact on workers’ willingness to stay in the workforce.

**Hypothesis 6** **(H6).** *Job satisfaction has a significant effect on the willingness to stay in the workforce*.

A good working environment can provide comfort [25], so as to realize workers’ potential [26] and increase the positive work perception. The better the positive perception, the higher the job satisfaction and the better the organizational commitments can be fulfilled. [28]. Therefore, it is believed that job satisfaction and willingness to stay in the workforce have a significant impact. Therefore, it is assumed that job satisfaction has a significant impact on the willingness to stay in the workforce.

**Hypothesis 7** **(H7).** *Physical and mental health has a significant effect on the willingness to stay in the workforce.*

Medical work that is a high-tech and high-intensive occupation, plus the impact of the pandemic, which has increased the pressure from the working environment, has affected medical workers’ physical and mental health, reduced their work concentration and confidence [10,11,13,14,15], and influenced their efficiency and the quality of medical care [30]. However, good physical and mental health can help them develop their endurance, enhance their concentration, work effectiveness, self-confidence, and thus increase their willingness to stay in the workforce [30,31,32]. Therefore, it is believed that the physical and mental health has a significant impact on the willingness to stay in the workforce.

### 3.3. Research Subjects

Scholars believe that the number of questionnaires for predictive analysis must be 3–5 times the largest number of the variables of an issue [47] and greater than 100 [48]. The number of formal questionnaires cannot be less than 100 [49], and when the parameter is greater than 2500, representativeness can be obtained [50] by getting more than 200 [51].

Although Taiwan has taken effective preventative measures against the pandemic from January 2020 to March 2021, due to the government’s decision-making errors and misjudgments, coupled with the shortage of vaccines, the COVID-19 began to get out of control from April 2021, and domestic community-acquired infections spread. Therefore, a six-month-long Taiwan-wide Level 3 epidemic alert was initiated. During this period, due to the rapid increase in the risk of the COVID-19 and the number of patients, plus the existing workload, a massive exodus of medical workers and social volume began to appear, causing the government and relevant units to pay attention. In the light of that, the researchers began to consult about the willingness to be interviewed from June to July 2021 and collect questionnaires, simultaneously. During the survey, the questionnaire data collection was delayed because the medical units and healthcare workers have been busy with patients and the risk of infection. Finally, a sample of 300 health care workers was selected for the study by intentional sampling. A total of 250 questionnaires were collected, with a response rate of 83.3%, and the number of valid questionnaires was 208, with an effective questionnaire response rate of 83.2%. The survey was carried out to verify the correlation between leisure barriers, job satisfaction, physical and mental health, and willingness to stay in the medical team.

### 3.4. Research Tools

The questionnaires in this study were modified and compiled with reference to relevant literature and questionnaires on leisure obstacles [18,20,40,41,42,43,44,45,46], job satisfaction [22,23,24,25,26,27,28,29], physical and mental health, and willingness to stay in the workplace [27,31,32]. The questionnaire consisted of 4 sections with a total of 40 questions: 2 questions on basic personal information on gender (male and female) and age (under 30, 31–40, 41–50, 41–60, 61+), 12 questions on leisure obstacles (personal, 4 questions; interpersonal, 4 questions; structural, 4 questions), 8 questions on job satisfaction (internal, 4 questions; external, 4 questions), 14 questions on physical and mental health (psychological feelings, 5 questions; mental state, 3 questions; life attitude and health, 6 questions), and 4 questions on willingness to stay in the workplace. The questionnaire was based on a five-point Likert scale, with each question rated from 1 to 5 on a scale of “strongly disagree” to “strongly agree”.

### 3.5. Data Processing and Analysis

After collecting the questionnaires and excluding the invalid questionnaires, the data were analyzed using IBM SPSS Statistics for Windows, Version 26.0 statistical software. The basic statistical tests and *t*-tests were used to analyze the perceptions of medical workers on leisure obstacles, job satisfaction, physical and mental health, and willingness to stay in the workplace, and then IBM AMOS statistics for Windows, Version 24.0 statistical software was used to analyze the correlation between the variables.

### 3.6. Limitations and Ethics Statement

Due to the risk of infection and the intense workload of health workers under the epidemic, as well as financial, time, physical, and human resource constraints, the researchers used an online questionnaire platform to collect the sample, which may have limited the number of samples collected and the target population. Respondents in this study were all professionals. The survey and questionnaire were designed in an anonymous manner with clear and explicit descriptions of the research questions, methods, and explicit and clear question design. Respondents were consulted about their willingness to be interviewed and their right to use the questionnaire data. In addition, the study was conducted by questionnaire and analyzed by statistical software without physical experimentation. Therefore, since the data were obtained adhering to strict confidentiality and with the consent of the respondents, the study considered all processes to be ethical.

## 4. Result Analysis

### 4.1. Sample Background Analysis

The valid sample for this study was 208 medical workers. In terms of gender, there were more females (112), accounting for 53.6% of the valid sample, and fewer males (96), accounting for 45.9% of the valid sample; in terms of age, a total of 76 people aged 31–40 years were the most numerous, accounting for 36.4% of the valid sample, while a total of 19 people aged 51–60 years or older were the least numerous, accounting for 9.1% of the valid sample, as shown in Table 1.

### 4.2. Difference Analysis

The medical workers’ perceptions of leisure obstacles, job satisfaction, physical and mental health, and willingness to stay in the workforce were statistically analyzed using the Arithmetic mean and *t*-test methods, and hypotheses 1–4 were tested. Analysis indicated that, among the leisure obstacles, requiring too many skills (2.27), lack of time (2.78), and lack of leisure information (2.48) scored higher, while family members’ lack of support (2.19), lack of transportation (2.50), and limited space (2.41) scored lower. In terms of job satisfaction, satisfaction with salary (3.39) and being able to do the job (3.76) scored higher, while promotion opportunities (3.19) and gaining a sense of accomplishment (3.60) scored lower. In terms of physical and mental health, less enthusiasm (3.02), mental weakness (2.87), and easily angered (2.68) scored higher, while reduced ability (2.53), frequent headaches (2.64), and irregular diet (2.61) scored lower. In terms of willingness to stay in the workplace, being able to use what you have learned (3.62) scored the highest, and being able to express your true self and work freely (3.45) scored the lowest. Moreover, on issues such as lack of family support (2.00:2.35), requiring too many skills (2.01:2.93), expensive equipment (2.43:2.74), no transportation (2.34:2.63), not having enough physical strength (2.44:2.79), no money (2.32:2.53), limited leisure space (2.21:2.58), promotion opportunities (2.84:3.49), being able to do the job (3.60:3.90), and meaningfulness of the job (3.68:3.81), there were significant differences (*p* < 0.01) in the perceptions of different genders, and women felt more strongly than men. The results of this analysis were inconsistent with the study hypotheses 1–4. As shown in Table 2.

### 4.3. Offending Estimates Tests

As shown in Table 3, the error variances in this study ranged from 0.01 to 0.05, and the standardized coefficients ranged from 0.50 to 0.83, which did not exceed 0.95. This result meets the criteria of detecting the presence of negative error variances and detecting whether the standardized regression coefficients exceed or are too close to 1 in the test of offending estimates [52]. Therefore, there is no violation of the estimation in the results data, and the overall model fit can be examined.

### 4.4. Offending Estimate

The reliability and validity of the questionnaire were examined by CFA and the questions were revised according to the Modification Indices (M.I.) [53]. In total, the questions were removed from the Personal 1 and Structural 4 of the Leisure Obstacles Scale; Internal 1 and External 1 of the Job Satisfaction Scale; Psychological 2, Psychological 5, Attitude 1, Attitude 2, Attitude 5, Attitude 6 of the Physical and Mental Health Scale, and Item 2 of the Willingness to Stay in the Workforce Scale.

#### 4.4.1. Verification of Convergent Validity

The composite reliability (C.R.) and average variance extracted (AVE) measures of questionnaire constructs can be used to determine the convergent validity [54]. The C.R. value should be greater than 0.6 and the AVE value should be greater than 0.5 for good convergent validity of the questionnaire [55]. The convergent validity of this study was examined for the components of leisure obstacles, job satisfaction, physical and mental health, and willingness to stay in the workforce, and the negative factor loadings for all components were between 0.68 and 0.91, with C.R. values between 0.89 and 0.96 and AVE values between 0.65 and 0.76, which met the proposed criteria for convergent validity, indicating good convergent validity of this study’s questionnaire [52,54,55,56], as shown in Table 4.

#### 4.4.2. Structural Model Analysis

In this study, the overall model fit was examined with reference to structural model analysis, using seven indicators such as the chi-square value (χ^2^), the ratio of χ^2^ to degrees of freedom, GFI, AGFI, RMSEA, CFI, and PCFI [53]. The smaller the ratio of χ^2^ to its degrees of freedom, the better [44], and the corrected ratio was 2.54 in this study. The closer the values of GFI and AGFI are to 1, the better [53], and the corrected values of GFI and AGFI were 0.80 and 0.73, respectively, in this study. The best RMSEA value is less than 0.08 [57], and the corrected RMSEA value in this study was 0.08. The standard value of CFI should be greater than 0.90, and the revised CFI in this study was 0.90. The PCFI should be at least 0.50, and the revised PCFI in this study was 0.79. The results of this study showed that the overall fitness indexes met the standard, as shown in Table 5.

**Figure 2:**
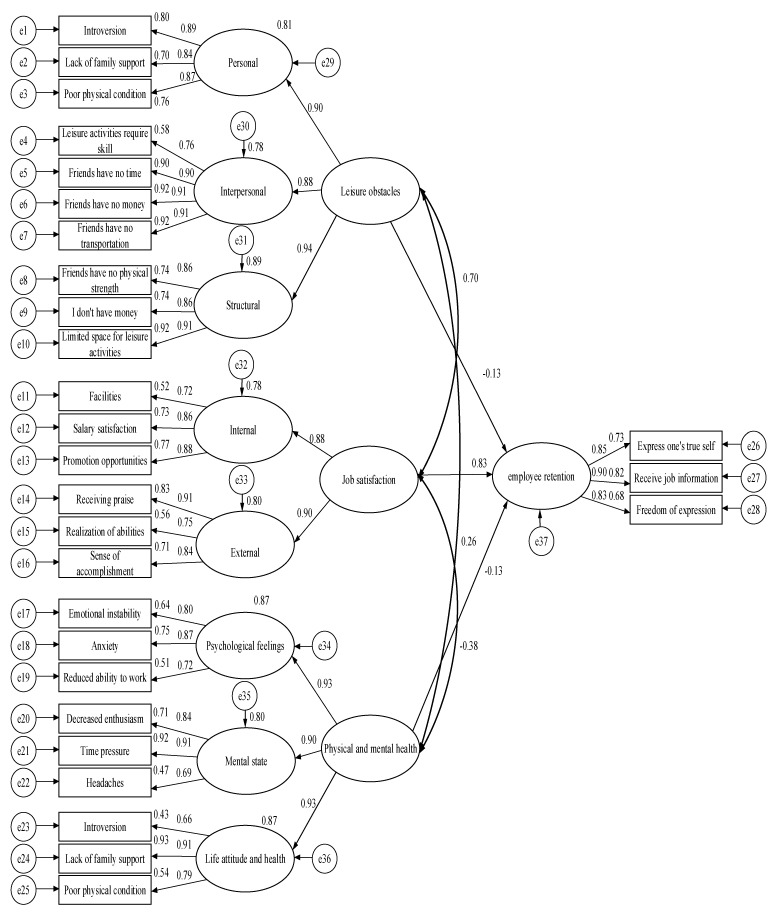
A model for leisure obstacles, job satisfaction, physical and mental health, and willingness to stay in the workforce among medical workers.

Structural model analysis was used to examine the perceptions of leisure obstacles, job satisfaction, physical and mental health, and willingness to stay in the workforce, and to test hypotheses 5–7. As shown in Figure 2, the analysis revealed that there were significant effects (*p* < 0.05) between leisure obstacles, job satisfaction, physical and mental health, and willingness to stay in the workforce. Among them, leisure obstacles, physical and mental health, and willingness to stay in the workforce were negatively correlated, and job satisfaction was positively correlated, as shown in Table 6. It can be seen that there was a correlation be-tween medical workers’ perceptions of leisure obstacles, job satisfaction, physical and mental health, and willingness to stay in the workforce. The higher the job satisfaction, the higher the willingness to stay in the workforce, while the higher the influence of leisure obstacles and physical and mental health status, the greater the willingness to leave the workforce. The results of this analysis are consistent with hypotheses 5–7 of the study.

### 4.5. Discussion

#### 4.5.1. Background and Limitations

In order to treat patients, medical care requires patience, love, and high concentration in addition to professional skills, so most medical workers are women. However, the impact of the pandemic, the increase in the number of people infected with the COVID-19, and the workload of treating the existing medical patients have deterred the young medical workers, and workers over the age of 51 have not been physically strong enough to cope with the heavy workload.

However, during the research and survey, Taiwan was facing an unprecedented crisis, and community-acquired infections have begun to appear [10,11,14]. The nation-wide health care units and medical workers were facing serious challenges, which have reduced their willingness to grant interviews and decreased the sample size analyzed in this study. Although the number of questionnaires is sufficiently representative, and the analysis was conducted [45,46,47,48,49], if we can resume the survey in the future, or increase the sample size and interview the medical workers in different medical and nursing positions, we will get more detailed and in-depth answers.

#### 4.5.2. Analysis of Medical Workers’ Perceptions of Leisure Obstacles, Job Satisfaction, Physical and Mental Health, and Willingness to Stay in the Workforce

Although relevant studies have confirmed that leisure activities can promote individual physical and mental health [18], currently medical workers’ workload have increased due to factors such as scheduling, professional work, techniques, and doctor-patient responsibilities [8,9,24], as well as the impact of the pandemic. As a result, all of these have hindered them from planning leisure activities [8,10,33], increased the pressure on the healthcare environment and work [8,10,11], affected healthcare workers’ physical and mental health [26,30], impacted their willingness to stay in the workforce [26,29,30,31], and they surely have the same perceptions. However, the study found that healthcare workers’ leisure activities have been hindered due to the lack of time, techniques and leisure information. Secondly, although they earn good salaries and their work is neither light nor hard, their job satisfaction has been reduced due to lack of promotion opportunities and sense of accomplishment. So, their enthusiasm for work has decreased, and their mental weakness and irritability led to delicate physical and mental health. As a result, they have been unable to perform tasks effectively and express themselves, and entertained the thought of quitting their jobs. This is consistent with neither the results described in the literature nor the Hypotheses 1–4 proposed by the research.

In our opinion, the current medical workers are mainly female, working long and irregular hours, and the shift system leads to irregular work and rest schedule, resulting in rapid staff turnover and slow replacement of human resources. In addition, the risk of infection has not yet been eliminated, and the number of infected patients may increase at any time due to the high risk of infections among the public. In addition, the nature of medical care, as well as the limitations of equipment, patients and hospital beds, do not allow for suitable leisure space and facilities in the work environment. As a result, medical workers face uncertain work pressure and environmental constraints under the epidemic, as well as structural differences between men and women in terms of physical fitness, endurance, and stamina, resulting in longer working hours, increased stress, and no leisure time, which can easily lead to physical overload, health crises, and a desire to leave the workforce.

Thus, medical workers believe that engaging in leisure requires too much skill, lack of time, and lack of information about leisure are the main reasons for the current leisure obstacles, while lack of promotion opportunities and low job satisfaction are factors in decreasing job satisfaction. As a result, medical workers feel less enthusiastic, less energized, emotionally angry, less free to work, less able to express their true selves, and have more thoughts of leaving their jobs, which women feel more strongly than men.

#### 4.5.3. A Model for Leisure Obstacles, Job Satisfaction, Physical and Mental Health, and Willingness to Stay in the Workforce among Medical Workers

Scholars believe that elimination of leisure constraints [19], reduction of negative emotions and problems of work tasks [27,28], and improvement in health [26,27,28,29,30,31], have a positive and significant impact on work efficiency and willingness to stay in the workforce [19,26,27,28,29,30,31]. According to the results of the analysis, there was a significant correlation between leisure obstacles, job satisfaction, physical and mental health, and willingness to stay in the workforce, which is consistent with the hypotheses 5–7 of the study.

We believe that although medical workers are physically active in their work and the environment is full of crises that create work stress, leisure can help to improve people’s physical and mental health, and the better their physical and mental health, the better they are able to cope with all kinds of stress and to feel confident in their work. The stronger the self-confidence of medical professionals in the workplace, the more stable their performance, the more effective their work is, and the more they can identify with their current behaviors. Therefore, if we can maintain reasonable and sufficient time for leisure activities and improve our physical and mental health, it will help people face the burden and pressure of life and work, which will, in turn, relieve or reduce the negative psychological state, create a sense of recognition for work and increase the desire to stay in the workforce.

Therefore, there is a correlation between medical workers’ perceptions of leisure obstacles, job satisfaction, physical and mental health, and their willingness to stay in the workforce, and the higher the degree of job satisfaction, the higher the willingness to stay in the workforce, while the higher the influence of leisure obstacles and physical and mental health, the greater the willingness to leave the workforce.

## 5. Conclusions

Because of the excessive skills required to engage in leisure, the lack of time and the lack of leisure information led to leisure obstacles for current medical workers. In the workplace, there are few opportunities for advancement, and job satisfaction is low due to low job achievement. In addition, most health workers have physical health problems such as reduced enthusiasm, mental weakness, and emotional irritability. As a result, they are unable to work freely, express their true selves, and have thoughts of leaving their jobs. There are gender differences in perceptions of the issues of leisure obstacles such as family support, skills, expensive equipment, friends’ lack of transportation and adequate physical strength, lack of personal funds, narrow leisure space, and the issues of willingness to stay in the workforce such as promotion, competence, and meaningfulness of work, and women feel more strongly than men. In addition, the higher the job satisfaction, the higher the desire to stay in the job, and the higher the influence of leisure obstacles and physical and mental health status, the greater the desire to leave the job.

Based on the conclusions, the following recommendations are made:

1. For policy

Central and local governments should pay attention to the crisis of medical staff attrition and provide a safe and secure work quality and environment for medical workers in service during the epidemic. Simultaneous planning of recreational spaces and design of short, effective, and low-cost recreational sports measures should be implemented to promote the physical and mental health of medical workers.

2. For institutions

Medical institutions should plan appropriate leisure space for medical staff in the hospital or in combination with the surrounding leisure sports venues.

3. For medical workers

Medical workers should be encouraged to spontaneously engage in leisure sports to relieve stress and ameliorate physical and mental health problems.

4. Recommendations for future research

Short-term, effective, and low-cost leisure exercise measures can be designed to promote the physical and mental health of health care workers. Based on the deficiencies of this research, future research can increase the number of samples, or adopt different research methods, research areas, and different medical departments for verification and investigation.

## Figures and Tables

**Table 1:** Descriptive statistics of subjects.

Issue	Options	N	%
Gender	Male female	96	45.9
		112	53.6
Age	Under 30	52	24.9
	31–40	76	36.4
	41–50	61	29.2
	51–60	19	9.1
	Over 61	52	24.9

**Table 2:** Analysis of the differences in perceptions of medical workers regarding leisure obstacles, job satisfaction, physical and mental health, and willingness to stay in the workplace.

Issue			N 208			Gender		*p*
			N	SD	Rank	Male	Female	
Leisure obstacles	Personal	Introversion	2.19	0.952	3	2.00	2.35	0.000 **
		Lack of family support	2.23	1.003	2	2.08	2.35	0.016
		Poor physical condition	2.27	0.945	1	2.01	2.49	0.000 **
	Interpersonal	Leisure activities require skill	2.78	1.103	1	2.60	2.93	0.187
		Friends have no time	2.60	1.068	3	2.43	2.74	0.000 **
		Friends have no money	2.50	1.012	4	2.34	2.63	0.001 **
		Friends have no transportation	2.63	1.104	2	2.44	2.79	0.002 **
	Structural	Friends have no physical strength	2.43	1.010	2	2.32	2.53	0.001 **
		I don’t have money	2.41	0.943	3	2.21	2.58	0.003 **
		Limited space for leisure activities	2.48	0.997	1	2.27	2.65	0.014
Job satisfaction	Internal	Facilities	3.39	0.977	1	3.30	3.47	0.412
		Salary satisfaction	3.19	1.055	3	2.84	3.49	0.008 **
		Promotion opportunities	3.38	0.985	2	3.13	3.60	0.059
	External	Receiving praise	3.60	0.873	3	3.48	3.71	0.062
		Realization of abilities	3.76	0.753	1	3.60	3.90	0.000 **
		Sense of accomplishment	3.75	0.765	2	3.68	3.81	0.005 **
Physical and mental health	Psychological feelings	Emotional instability	2.57	0.970	2	2.44	2.69	0.327
		Anxiety	2.53	0.942	3	2.58	2.49	0.134
		Reduced ability to work	3.02	1.088	1	3.13	2.94	0.017
	Mental state	Decreased enthusiasm	2.64	0.911	3	2.64	2.64	0.031
		Time pressure	2.87	0.962	1	2.91	2.84	0.136
		Headaches	2.69	0.912	2	2.77	2.63	0.127
	Life attitude and health	Mental weakness	2.61	1.049	2	2.67	2.55	0.031
		Hypersensitivity to pain	2.68	0.893	1	2.68	2.69	0.037
Willingness to stay in the workforce		Express one’s true self	3.45	0.976	2	3.29	3.58	0.469
		Receive job information	3.45	0.931	2	3.14	3.72	0.210
		Freedom of expression	3.62	0.876	1	3.49	3.73	0.099

** *p* < 0.01.

**Table 3:** Offending estimates tests for the leisure obstacles scale.

Item		Standardized Regression Coefficient	Error Variance
Leisure obstacles	Introversion u ← Individual 1	0.81	0.04
	Lack of family support ← Individual 2	0.89	0.03
	Poor physical condition ← Individual 3	0.83	0.04
	Leisure activities require skill ← Individual 4	0.88	0.03
	Friends have no time ← Individual 1	0.76	0.06
	Friends have no money ← Individual 2	0.89	0.03
	Friends have no transportation ← Interpersonal 3	0.91	0.03
	Friends have no physical strength ← Interpersonal 4	0.91	0.03
	I don’t have money ← Structural 1	0.88	0.03
	Limited space for leisure activities ← Structural 2	0.84	0.03
	I lack leisure information ← Structural 3	0.90	0.03
	I lack transportation ← Structural 4	0.82	0.04
Job satisfaction	Facilities ← Internal 1	0.78	0.04
	Salary satisfaction ← Internal 2	0.75	0.05
	Promotion opportunities ← Internal 3	0.86	0.04
	Receiving praise ← Internal 4	0.85	0.04
	Realization of abilities ← External 5	0.94	0.02
	Sense of accomplishment ← External 1	0.91	0.02
	Competent in the workplace ← External 2	0.74	0.03
	Meaningfulness ← External 3	0.77	0.03
Physical and mental health	Emotional instability ← Psychological feelings 1	0.82	0.04
	Anxiety ← Psychological feeling 2	0.87	0.03
	Reduced ability to work ← Psychological feeling 3	0.85	0.03
	Decreased enthusiasm ←Psychological feeling 4	0.69	0.07
	Time pressure ← Psychological feeling 5	0.65	0.06
	Headaches ← Psychological feeling 6	0.84	0.03
	Mental weakness ← Mental state 1	0.90	0.03
	Hypersensitivity to pain ← Mental state 2	0.71	0.04
	Insomnia ← Mental state 3	0.71	0.06
	Indigestion ← Life attitude and health 1	0.79	0.04
	Irregular eating ← Life attitude and health 2	0.67	0.06
	Irritability ← Life attitude and health 3	0.84	0.03
	No confidence ← Life attitude and health 4	0.83	0.03
	Suicidal mentality ← Life attitude and health 5	0.61	0.05
Employee retention	Express one’s true self ← Willingness to stay in the workforce 1	0.83	0.04
	Receive job information ← Willingness to stay in the workforce 2	0.80	0.02
	Freedom of expression ← Willingness to stay in the workforce 3	0.91	0.03
	Use what one has learned ← Willingness to stay in the workforce 4	0.84	0.03

**Table 4:** Validation analysis of leisure hindrance, job satisfaction, physical and mental health, and willingness to stay in the workforce.

Perspective	Index	Standardized Factor Loading	Non-Standardized Factor Loading	S.E.	C.R. (t-Value)	*p*	SMC	C.R.	AVE
Leisure obstacles	Introversion	0.89	1.00				0.80	0.96	0.76
	Lack of family support	0.84	0.99	0.06	16.19	***	0.70		
	Poor physical condition	0.87	0.97	0.06	17.32	***	0.76		
	Leisure activities require skill	0.76	1.00				0.58		
	Friends have no time	0.90	1.14	0.08	14.01	***	0.80		
	Friends have no money	0.91	1.09	0.08	14.02	***	0.82		
	Friends have no transportation	0.91	1.20	0.08	14.22	***	0.82		
	Friends have no physical strength	0.86	1.00				0.74		
	I don’t have money	0.86	0.95	0.06	15.98	***	0.74		
	Limited space for leisure activities	0.91	1.06	0.06	17.64	***	0.82		
Job satisfaction	Facilities	0.72	1.00				0.52	0.92	0.68
	Salary satisfaction	0.85	1.28	0.11	11.67	***	0.73		
	Promotion opportunities	0.88	1.22	0.11	11.3	***	0.77		
	Receiving praise	0.91	1.00				0.83		
	Realization of abilities	0.75	0.71	0.06	12.62	***	0.56		
	Sense of accomplishment	0.84	0.81	0.05	15.49	***	0.71		
Physical and mental health	Emotional instability	0.80	1.00				0.63	0.94	0.65
	Anxiety	0.88	1.07	0.08	13.53	***	0.77		
	Reduced ability to work	0.71	1.00	0.09	10.58	***	0.51		
	Decreased enthusiasm	0.84	1.00				0.70		
	Time pressure	0.91	1.15	0.07	15.38	***	0.82		
	Headaches	0.69	0.83	0.07	11.16	***	0.48		
	Introversion	0.68	1.00				0.47		
	Lack of family support	0.87	1.08	0.10	11.04	***	0.76		
	Poor physical condition	0.84	1.07	0.10	10.97	***	0.71		
employee retention	Express one’s true self	0.86	1.00				0.74	0.89	0.74
	Receive job information	0.90	1.01	0.07	15.46	***	0.81		
	Freedom of expression	0.83	0.87	0.06	14.28	***	0.68		

*** *p* < 0.001.

**Table 5:** Analysis of the overall model fit.

Fit Index	Tolerable Range	Modified Model	Model Fit Determination
χ^2^ (Chi-square)	The smaller the better	786.72	
χ^2^ anddegree of freedom ratio	<3	2.54	Fit
GFI	>0.80	0.80	Fit
AGFI	>0.80	0.73	same
RMSEA	<0.08	0.08	Fit
CFI	>0.90	0.90	Fit
PCFI	>0.50	0.79	Fit

**Table 6:** Test results of the study hypotheses.

Hypothesis	Path Relationship	Path Value	Hypothesis True/False
5	Leisure obstacles → Willingness to stay in the workforce	−0.13 *	True
6	Job satisfaction → willingness to stay in the workforce	0.83 *	True
7	Physical and mental health → willingness to stay in the workforce	−0.13 *	True

Source: Compiled from this study * *p* < 0.05.

## Data Availability

No specific data were used to support this study.
